# The Engineering Side of Hospital Work

**Published:** 1905-03-18

**Authors:** 


### THE ENGINEERING SIDE OF HOSPITAL WORK.

*Continued from page* 429.

### VII.—DISINFECTORS AND STERILISERS.

As indicated in a previous article, disinfecting, the destruction of bacilli, and their removal from clothes, bedding, etc., may be accomplished by the aid of hot air or steam, though the latter is rapidly becoming the general favourite. Hot-air disinfectors are, however, made and used. One that is made by Messrs. Summerscales and Sons of Keighley, is constructed as follows. A brick chamber is built over a pit to which steps are arranged. The chamber is fitted with a chimney and two iron doors on opposite sides, with rails leading through the chamber for the wagons with the infected material to run on. In the pit under the chamber is a furnace, which is lighted when the disinfector is in operation. The wagon containing the infected articles is wheeled into the chamber, the doors closed, and the inside of the chamber submitted to a heat of from 260° to 300° F. for one or two hours, according to the nature of the articles to be treated. At the end of that period a valve is opened between the chamber and a flue arranged to be maintained at a high temperature, where the fumes from the articles, etc., are cremated, something in the same way as in the hot flue of the refuse destructor. When the fumes have all been consumed, the door opposite to that at which the wagon entered, is opened, the wagon run out, and another run in, if required. The process is claimed to be simple and efficient. Where there is not space for running the wagon out by a second door, the chamber can be built with a single door, the articles going in and out the same way. It is obvious that many modifications of this arrangement are practicable.

At the Westminster Hospital they have an apparatus on somewhat similar lines. It consists of a brick chamber, fitted with shelves, on which trays rest containing the clothes to be disinfected. There is a flue at the back of the chamber, with a small furnace, the heat from the furnace gases warming the chamber. The clothes remain in the apparatus six hours. The fire is controlled by a damper.

At King's College Hospital they have a chamber on somewhat similar lines heated by gas, the fumes being carried off by a flue in the wall.

### Steam-Heated Disinfectors.

These are very generally used, and are all constructed on certain main lines. There are usually two contiguous rooms situated in the basement, one for receiving the infected clothes, and the other for delivering them when they have been disinfected; and the disinfecting chamber, which is of iron or steel, is built into the wall separating the two. Messrs. Manlove and Alliott, of Nottingham, who have done a very large amount of this work, recommend that the disinfector, when in its place in the wall, shall be covered with a layer of a thermal non-conductor, such as silicate cotton, and the wall built close down on this, the thermal insulator providing the necessary room for expansion. The disinfecting chamber is sometimes cylindrical, sometimes rectangular but more frequently elliptical in section, and is usually fitted with rails or runners, and a cage to hold the articles to be disinfected, the cage running into the chamber on the rails by a door at one end, and out by a door at the other end, the two doors being on opposite sides of the dividing wall. The chamber is virtually an oven. It nearly always has a jacket, or space surrounding its whole length, into which steam can be passed, the steam being used to heat the chamber. In some forms the chamber is warmed by steam passing in a coil of pipe in the chamber itself. In all cases, however, it is arranged to admit steam to the chamber, as well as to the jacket. In the early form of the Washington-Lyon apparatus, upon which nearly every apparatus upon the market has been based, only steam to the jacket, and steam to the chamber were provided, the chamber being first warmed by the admission of steam to the jacket, then the clothes put in the chamber, steam admitted to the chamber, the clothes allowed to remain for a certain time, the steam shut off, and the clothes dried by subjection to the continuous application of heat by the jacket steam. Two important improvements have been made in the apparatus, in comparatively recent years. Steam of higher pressure is now employed, and Messrs. Manlove and Alliott's Vacuum attachment has been added. In the writer's opinion it would be difficult to exaggerate the importance of the latter. By higher pressure steam must be understood steam a few pounds above the pressure of the atmosphere, but not having its connection with the boiler severed. Saturated steam, as explained in a former article appears to have certain virtues which superheated steam has not. Saturated steam contains a certain quantity of the vapour of water, and this is condensed on the clothes to be disinfected, and later on is re-evaporated, the re-evaporation being of great assistance in drying the goods after the disinfecting process is complete. As mentioned in connection with refuse destructors, the condensation and re-evaporation of the moisture carried over from the boiler with the steam is often a valuable aid to the engineer, though in other conditions it gives him a good deal of trouble. Steam, then, at about 260° Fahr., or at the absolute pressure of 36 lbs. per square inch, is used for disinfecting. This pressure corresponds to about 21 lb. above the pressure of the atmosphere. Steam at this pressure, provided it is not too heavily charged with the vapour of water, is found to be very efficient in disinfecting. It will penetrate to the centre of such things as mattresses, blankets, and woollen clothes, which offer a high resistance to the passage of heat, and while the moisture it carries is often condensed within the substance, the re-evaporation exercises a beneficial effect. The temperature is found absolutely sufficient to kill all known bacilli. In fact a lower temperature will do this, but the higher temperature ensures the penetration of the heat to the innermost interstices of the fabrics. Another point in favour of steam at the pressures that have been named, is the fact that a much shorter time is sufficient for disinfecting than with hot air.

It should be mentioned that superheated steam, steam that has been deprived of all its moisture, and reduced to the condition of a gas, pure and simple, appears to act very much as hot air does. It will disinfect, but it requires a considerable time, just as hot air does, and it is simpler to use heated air than superheated steam.

The other improvement mentioned, the addition of the vacuum attachment, is a further great advance. The *modus operandi* where this arrangement is fixed is as follows: the chamber is first warmed, as before, the clothes to be treated are put in, a vacuum is then created by exhausting the air from the chamber to 10 to 12 barometric inches below ordinary atmospheric pressure. This arrangement draws the air out of the interstices of the clothing, and prepares the way for the steam to enter. After a certain time steam is admitted to the chamber, the steam having been dried by passing through a steam-heated coil on its way to the chamber, and it being thereby assured that there shall not be an excess of moisture present in the steam. An excess would lead to trouble in other ways, the colours of the fabrics might run, and generally there would be more water present than is wanted. After a certain time the steam is turned off, and warmed air is passed into the chamber, the air being warmed by passing through a similar arrangement to that which dries the steam. After a further interval the door in the room on the opposite side to that at which the clothes entered is opened and the clothes withdrawn quite dry. Twenty minutes has been found sufficient for the clothes to be subjected to the full pressure of the steam for complete disinfection to take place. Another modification of the steam disinfector is made by Messrs. Thos. Bradford and Co., the well-known laundry engineers. It is practically the hot-air apparatus of Messrs. Summerscales, adapted for steam, with certain improvements of its own. There is a rectangular chamber of wrought iron, constructed with a jacket, as explained, and the chamber is fixed over a pit, just as Messrs. Summerscales' hot-air chamber is. There is a boiler and furnace in the pit. The steam from the boiler passes through a pipe in the bottom of the chamber into the jacket, while the gases which are formed in the process of combustion pass immediately under the iron plate forming the bottom of the chamber on their way to the flue, through which they escape to the atmosphere. At the top of the chamber there is a damper, closing a passage to the boiler flue, and at the bottom of the chamber there is a second passage to the boiler flue. The steam, as it passes over the hot plate forming the base of the chamber, is superheated to a small extent. After it has passed through the jacket it enters the chamber at the top, passes down through the clothes in the chamber, and out to the flue. When the clothes to be disinfected have been in the chamber a sufficient time, the damper at the top of the steam jacket is opened, allowing the steam to pass directly to the flue by the upper flue pipe. The heat of the jacket then, it is stated, drives off all moisture from the clothes, in a similar manner to the apparatus already described. It is stated that the time required for disinfecting each batch of articles is 30 minutes, and the time from the lighting of the fire, required for preparing the apparatus for use, is less than an hour. The chamber is first heated by the jacket steam, the clothes put in, and the steam turned through them. It will be observed that the apparatus is independent of the boiler service of the hospital, so that it can be placed in any position where it would be troublesome to carry steam pipes from the boilers.

Messrs. Manlove and Alliott make a self-contained apparatus on the lines of their apparatus described above, with its own boiler, for cases where it is inconvenient to carry steam from the boiler.

*(To be continued.)*

